# L.U.St: a tool for approximated maximum likelihood supertree reconstruction

**DOI:** 10.1186/1471-2105-15-183

**Published:** 2014-06-12

**Authors:** Wasiu A Akanni, Christopher J Creevey, Mark Wilkinson, Davide Pisani

**Affiliations:** 1 Department of Biology, The National University of Ireland, Maynooth, Maynooth, Kildare, Ireland; 2 Department of Life Sciences, The Natural History Museum, London SW7 5BD, UK; 3 Institute of Biological, Environmental and Rural Sciences (IBERS), Aberystwyth University, Aberystwyth, Ceredigion SY23 3FG, UK; 4 School of Biological Sciences and School of Earth Sciences, The University of Bristol, Woodland Road, BS8 1UG Bristol, UK

**Keywords:** Supertrees, Maximum likelihood, Phylogenomics, Tests of two trees

## Abstract

**Background:**

Supertrees combine disparate, partially overlapping trees to generate a synthesis that provides a high level perspective that cannot be attained from the inspection of individual phylogenies. Supertrees can be seen as meta-analytical tools that can be used to make inferences based on results of previous scientific studies. Their meta-analytical application has increased in popularity since it was realised that the power of statistical tests for the study of evolutionary trends critically depends on the use of taxon-dense phylogenies. Further to that, supertrees have found applications in phylogenomics where they are used to combine gene trees and recover species phylogenies based on genome-scale data sets.

**Results:**

Here, we present the L.U.St package, a python tool for approximate maximum likelihood supertree inference and illustrate its application using a genomic data set for the placental mammals. L.U.St allows the calculation of the approximate likelihood of a supertree, given a set of input trees, performs heuristic searches to look for the supertree of highest likelihood, and performs statistical tests of two or more supertrees. To this end, L.U.St implements a winning sites test allowing ranking of a collection of *a-priori* selected hypotheses, given as a collection of input supertree topologies. It also outputs a file of input-tree-wise likelihood scores that can be used as input to CONSEL for calculation of standard tests of two trees (e.g. Kishino-Hasegawa, Shimidoara-Hasegawa and Approximately Unbiased tests).

**Conclusion:**

This is the first fully parametric implementation of a supertree method, it has clearly understood properties, and provides several advantages over currently available supertree approaches. It is easy to implement and works on any platform that has python installed.

Availability: bitBucket page - https://afro-juju@bitbucket.org/afro-juju/l.u.st.git.

Contact: Davide.Pisani@bristol.ac.uk.

## Background

Supertree methods are generalisation of consensus methods to the case of partially overlapping input trees, and any method that can be used to amalgamate a collection of such trees is a supertree method [1]. Supertrees were formally introduced to the realm of the classification sciences by Gordon [2], who described a Strict Consensus Supertree method. However, the first supertree algorithm was introduced by Aho and colleagues [3] as an application to merge partially overlapping databases. Since these early works, there has been a lot of interest in supertree reconstruction particularly in evolutionary biology where supertrees have found an application as meta-analytical tools used to combine, and derive inferences from, published phylogenetic trees. Purvis [4] presented the first application of a supertree in this context merging primate phylogenies obtained from the literature to generate a supertree, and using it to test evolutionary hypotheses. Since then, the application of supertrees and more specifically their use for reconstructing large phylogenies in evolutionary biology has continued to be on the rise, paralleled by a substantial interest in the development of supertree methods. More recently, supertrees have also found important applications in genomics where they have been used to combine gene trees and derive species phylogenies [5-9].

A large number of supertree methods have been developed since the time of the Aho algorithm. However, most actual supertrees have been derived using the Matrix Representation with Parsimony (MRP) method of Baum [10] and Ragan [11]. This is due to the availability of excellent parsimony software and the general good understanding of the theory underlying parsimony. Yet theoretical justifications for the application of parsimony to the supertree setting are weak, and MRP is mostly implemented due to the fact that it is easily applicable in practice and tends to return well-resolved trees [12]. More generally, most available supertree methods are ad hoc, their properties being often poorly known, and the rationale for their application unclear [13-15]. The only exceptions seem to be those based on generalisations of well-known consensus methods [16], and the maximum likelihood (ML) method of Steel and Rodrigo [17].

We present a Python implementation of the ML supertree method of Steel and Rodrigo [17]. The method has been shown to be consistent on general statistical conditions unlike other approaches like MRP [17], and it is closely related to the majority rule (-) supertree method [16], with which it has been suggested to share important properties, in particular the fact that the supertrees it generates have been suggested to be, like those derived using majority rule (-), median trees for the input set [17].

The method is “approximate” in the sense that, likelihood vales are not normalised for tree size. However, it has been pointed out that at the least in the context of Maximum Likelihood analyses, given the parametric conditions under which our software is limited to work, this should not be a problem [18].

The ML supertree method is available as part of the Likelihood Utility for Supertrees (L.U.St) package. L.U.St is licensed under the GNU General Public License. Once downloaded, L.U.St can be run on any platform on which python is installed.

## Implementation

L.U.St’s estimation of the ML supertree operates by taking as input a file containing a set of newick-formatted trees (i.e. the input trees). L.U.St’s ML supertree method navigates the tree space using four alternative heuristic search strategies, varying in their speed and heuristic nature (these are compared elsewhere [19]). These are all based on Subtree Pruning Regrafting (SPR) algorithm. The user can either provide a starting supertree for the search or L.U.St can generate a random starting supertree using a stepwise addition technique. It should here be noted that as in standard ML phylogenetic analyses, providing a non-random starting tree (in the case of supertree reconstruction this could be a MRP supertree) would speed up the analysis. The likelihood score of the proposed supertree is calculated by first estimating the likelihood of each input tree, given the current supertree. After that, all input-tree wise likelihood values are summed to get the likelihood of the proposed supertree. Input tree wise likelihood values are calculated assuming that each input tree can be considered a subsample of the proposed supertree generated by pruning taxa and reconstructed with or without some topological distortion or incongruence. To calculate an input tree-wise likelihood value the proposed supertree is pruned to have the same taxon set of the considered input tree. After that the symmetric difference on full splits (i.e. the Robinson-Fould’s distance) [20], designated as *d*, between the pruned supertree and the input tree is calculated, in order to evaluate how dissimilar the input tree and the supertree are. The symmetric difference (*d*) is then used to calculate the input-tree likelihood using Steel and Rodrigo’s formula:

$${{\mathbb{P}}_{T}}_{,\;ϒ}\left[T^{'}\right]\;=\alpha \;\text{exp}\left[-\beta d\left(T^{'},T\left|ϒ\right)\right)\right]$$

Where α is a normalising constant and β is a value representing the quantity and quality of the data used to infer the input tree. An exponential distribution is used to model phylogenetic error. This implies that the probability that a given input tree is a sample of the proposed supertree decrease exponentially as *d* increases. The likelihood of each proposed superteee is then calculated summing across all tree-wise likelihood scores.

The method is “approximate” in the sense that, likelihood vales are not normalised for tree size. This means that the likelihood we calculate is a “weighted” sum of the input tree likelihoods, where the weights correspond to the tree-specific normalising constant (α). Albeit calculating these normalising factors is in theory possible [18], it is computationally very time consuming. However, Bryant and Steel [18] pointed out that if one uses small β values, the normalising constants simplify to a value that can be approximated using α = 1 irrespective of the input-tree sizes. For pragmatic reason (to maximise speed of execution), we currently do not allow the user to select β, has been fixed to a low value (β = 1). This should result in the normalising factor of (α), of Steel and Rodrigo [17] to simplify to a value of one (i.e. α = 1). It has been pointed out that at the least in the context of Maximum Likelihood analyses this should not affect the ranking of the supertrees [18]. Indeed analyses performed to test the accuracy of the method and to compare it with other supertree methods, seem to confirm Bryant and Steel results [19]. But we acknowledge that the ranking will be based on approximate, rather than correct, likelihood values.

L.U.St includes methods that allows for a variety of extra functions, including statistical tests for choosing between alternative hypotheses (tests of two trees – Winning site test, Kishino Hasegawa (KH) test [21], Shimidoara Hasegawa (SH) test [22] and the Approximately unbiased (AU) test [23]). Whilst the winning site test can be run natively in L.U.St, the calculation of KH, SH, AU and other tests requires the use of CONSEL [24]. To our knowledge there is no other software package that allows the extension of standard tests of two trees to the supertree framework. However, tests of two trees can have great utility in supertree research, as they can be used, for example, to investigate the extent to which current evidence (i.e. currently published trees) support alternative phylogenetic hypotheses (i.e. a set of proposed supertrees). Further to that, tests of two trees can be used in the phylogenomic context to evaluate the extent to which a set of gene-trees can reject a set of alternative phylogenetic hypotheses (i.e. a set of supertrees). Below an example of the use of test of two super(trees) in the phylogenomic context is provided.

L.U.St offers the user other useful functions to randomly resolve polytomies, deroot trees, reroot trees, resolve polytomies in a set of trees according to a user-provided input tree, create bootstrap replicates of input tree datasets, prune phyologenies, convert nexus formatted trees to the newick format and vice versa, and extract the taxon set of sets of trees.

### Example

Using supertree to investigate deep placental phylogeny.

**Figure 1:**
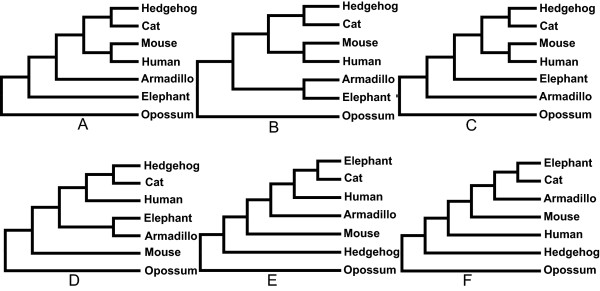
**The six compared mammal phylogenies. (A)** Afrotheria root; **(B)** Atlantogenata root; **(C)** Xenarthra root; **(D)** Rodentia root; **(E)** Hedgehog root hypothesis of [32]; **(F)** Hedgehog root hypothesis of [31].

Several hypotheses have been proposed for the position of the root of the placental mammals (Figure 1). Those that received the greatest support in recent studies are: (i) the “Xenarthra root” [25], which places the xenarthrans (i.e. armadillos, the anteaters, the tree sloths etc.) as the sister group to all the remaining placentals, (ii) the “Afrotheria root” [26,27], which places the Afrotheria (i.e. sea cows, manatees, aardvarks etc.) as the sister group to all the remaining placentals, (iii) the “Atlantogenata root” [28-30] suggesting that the sister group to the all the remaining placentals is is a clade comprising Afrotherian and the Xenarthrans. Further hypotheses that have historically been suggested include, for example (iv) the “hedgehog-1 root” placing the hedgehog (a Laurasiatherian) as the sister group of all the other placentals [31], (v) “hedgehog-2 root”, placing the hedgehog as the sister group of all the placentals followed by the rodents [32], and (vi) the “murids root” placing the mouse and the rat as the sister group of all the other placentals, and often finding the other rodents as a paraphyletic assemblage (e.g. [33], Figure 1A-F). Signals for the topologies in Figure 1A-B, and to a lesser extent Figure 1C, have been identified in many mammalian genes [27]. The fact that many different genes support different sets of relationships has resulted in a strong (still unresolved) debate about the correct placement of the root of the placental tree (contrast [25,27,30]). On the contrary, signal for the trees in Figure 1D-F is scant and these topologies most likely represent tree reconstruction artefacts (e.g. model misspecification [34], signal saturation [35], and long branch attraction [35,36]).

We decided to present an exemplar phylogenomic study of the mammalian relationships to illustrate our supertree software because, based on current knowledge, we can make predictions about what results to expect from our analyses and investigate whether the actualised outcomes from our software deviate from our expectations. More precisely, based on the results of [27] we expect that: (1) either the Afrotheria (Figure 1A) or the Atlantogenata (Figure 1B) hypotheses will emerge in our optimal ML supertree (most genes in mammalian genomes support one of these two topologies). (2) Similarly, a bootstrap majority rule consensus tree will most likely display one of the two above-mentioned hypotheses (Figure 1A or B). However, (3) as many genes are known to support both the topologies in Figure 1A-B (and to a lesser extent the tree in Figure 1C), bootstrap support for the basal placental split in the optimal ML supertree (and in the bootstrap consensus tree) are expected to be low. (4) Tests of two trees are not expected to be able to differentiate significantly between the topologies in Figure 1A-B. Indeed, given the results of [27] we can confidently predict that the trees in Figure 1A and B should be the first and second best fitting hypotheses, even though we cannot predict what their relative order will be (i.e. whether the tree in Figure 1A or in Figure 1B will be the best fitting one). Similarly, (5) whilst we cannot predict whether the Xenarthra hypothesis of Figure 1C will be significantly rejected by the Approximately Unbiased (or by another) test (e.g. Kishino-Hasegawa test), we can predict that this hypothesis should emerge as the third best one (see [27]). Finally, although we cannot make predictions about how the trees in Figure 1D-F will be ranked, given what is known of the distribution of the signal in mammal gene trees [27], we would expect all these hypotheses to be significantly rejected by the data and to emerge as the three hypotheses that worst fit our data.

To reconstruct our ML supertree of the placental mammals the gene-trees dataset of [9] was employed. This gene-trees data set was pruned to exclude irrelevant taxa using Clann [37]. Only 6 placentals (human, mouse, cat, hedgehog, elephant and armadillo) and one marsupial (the opossum) were retained. This meant that the dataset was reduced from 42 taxa overlapping on 2216 gene trees to 7 taxa overlapping on 389 gene trees (with the gene trees being partially overlapping and containing between 4 and 7 taxa).

## Result and discussion

**Figure 2:**
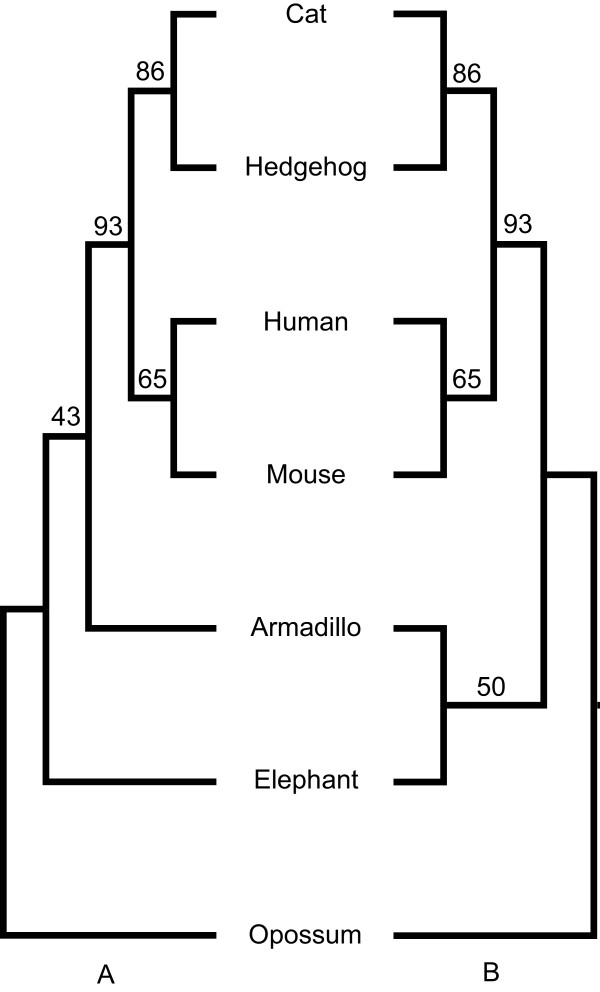
**Results of supertree analyses. (A)** Maximum likelihood supertree of the placental mammals. **(B)** Bootstrap Majority Rule Consensus Supertree.

L.U.St was used to estimate a placental ML supertree. The ML analysis was run for ten iterations with the heuristic search option set to 4 (i.e. using the fastest, least exhaustive, of the search strategies currently available in L.U.St). The pruned MRP supertree from [9] was used as starting tree. The resulting optimal ML supertree supports Afrotheria (Figure 2A). Twenty bootstrapped sets of trees were generated and ML supertree analyses were carried out for each to evaluate support for the inferred relationship of the placental mammals. A majority rule consensus was used to summarise the set of optimal supertrees from the bootstrap analyses and derive support values for the nodes in the optimal ML tree reported in Figure 2A. In addition to that we also report the Majority Rule consensus tree (Figure 2B), which differently from the optimal ML supertree, supports Atlantogenata. As expected (see above) the data provides almost equal support to Afrotheria and Atlantogenata (with the ML supertree supporting Afrotheria even though in the bootstrap replicates Atlantogenata was more frequently recovered). As expected trees representing other alternative hypothesis Xenarthra root (Figure 1C), murids root (Figure 1D), and the two hypotheses with a hedgehog root (Figure 1E and F) obtained lower (~6% bootstrap support for the Xenarthra and murid roots hypotheses) or no support (the hypotheses where the hedgehog was the sister group of all the other taxa). L.U.St was then used to estimate, for each one of the 389 input gene-trees, its tree-wise likelihood under each of the six alternative supertree topologies in Figure 1A-F. The input-tree-wise likelihood scores were then inputted into CONSEL to perform tests of two trees. The results from this analysis (Table 1) show that, as expected, the Approximately Unbiased test was not able to reject any of the three mainstream hypotheses (Afrotheria, Atlantogenata, and Xenarthra-root). Afrotheria emerged as the hypothesis that best fits the data (as expected given that it was represented in our optimal ML supertree), and as expected Xenarthra-root emerged as the third best-fitting hypothesis. Finally, also in this case in agreement with our expectations, all remaining hypotheses (Figure 1D-F) were significantly rejected by the data. Note that the more conservative Shimidoara-Hasegawa test was not able to reject the rodent basal hypothesis of Figure 1D. However, this test is well known to be over-conservative [23], hence also this result is essentially in line with our expectations.

**Table 1:** Results of the test of two trees

**Hypotheses**	**Approximate likelihoods**	**Ranks**	**AU test**	**SH test**	**KH test**
Afrotheria root	-487.092	1	0.628	0.886	0.579
Atlantogenata root	-487.960	2	0.496	0.874	0.421
Xenarthra root	-493.172	3	0.128	0.614	0.146
Muridae root	-523.573	4	0.001	0.017	0.003
Erinaceous root 1	-568.739	5	9E-08	0	0
Erinaceous root 2	-586.111	6	1E-07	0	0

Hypotheses tested are those from Figure 1.

All results generated were in agreement with our expectations (see above) and apart from confirming that the phylogenetic relationships of the mammals are still far from being resolved, they illustrate that L.U.St behave as expected and return results that reflect well current understanding of mammal evolution. Overall this illustrates that L.U.St will represent a useful tool in phylogenomics and supertree reconstruction more broadly.

## Conclusions

L.U.St represent the first implementation of a maximum likelihood supertree method. This method calculates approximate ML values and has the advantage of finding a tree that has been suggested might be representative of the median of the set of input trees when the symmetric difference metric is used to calculate the tree-to-tree distance. An added advantage of having an approximate ML supertree implementation is that it allows performing statistical test on trees to choose between alternative hypotheses. The results obtained with our toy example reflect current knowledge of mammalian evolution and confirm that the L.U.St package behaves as expected when used to attempt resolving a phylogenetic problem that is well known to be difficult. Being a freely available package for the Python programming environment, L.U.St is both flexible and platform-independent while also being user friendly and easy to implement.

## Availability and requirements

**Project name:** L.U.St.

**Project home page:**https://afro-juju@bitbucket.org/afro-juju/l.u.st.git.

**Operating system(s):** Linux.

**Programming language:** Python.

**Other requirements:** Consel.

**License:** GNU GPL.

## Competing interests

There were are no conflicting interests.

## Authors’ contributions

WAA and CJC implemented the software while WAA and DP conducted the experiments and WAA, DP and MW wrote the manuscript. All authors read and approved the final manuscript.
